# Features and outcomes of rectal cancer patients treated in a hospital in Bogotá, Colombia: a retrospective cohort study

**DOI:** 10.1038/s41598-023-41439-0

**Published:** 2023-09-08

**Authors:** Julián Andres Romo, Carlos Edgar Figueroa Avendaño, Laura A. López, Natalia Mesa, Alejandro González-Muñoz, David Baquero, Andrea Recamán, Fernando Rabeya, Alejandro Villabon, Isabella Velandia Sánchez, Álvaro Flechas

**Affiliations:** 1 Escuela de Medicina y Ciencias de la Salud, Universidad del Rosario, Bogota, Colombia; 2 Centro de Investigaciones de Méderi – CIMED, Hospital Universitario Mayor – Mederi, Bogota, Colombia

**Keywords:** Cancer, Gastrointestinal diseases

## Abstract

Rectal cancer is an increasing disease worldwide. The outcomes of its treatment are related to the preoperative characteristics of the patient. The objective of this study was to describe sociodemographic, clinical and surgical characteristics and outcomes of patients operated on for rectal cancer at Hospital Universitario Mayor Méderi (HUM) during the period within 2013–2017.A retrospective descriptive cohort-type study was carried out by consulting the clinical records of patients above the age of 18 years with a clinical/histopathological diagnosis of rectal cancer and an institutional follow-up in those who underwent surgery with laparoscopic anterior resection of the rectum carried out by the coloproctology service of the HUM between 2013 and 2017. For statistical analysis, the SPSS V22 program was used.Data from 133 patients were collected during the study period, most of them male, with more frequent involvement of the lower rectum. Complications occurred in 25% of the patients. Conversion rate to open surgery was 8.6%, in-hospital death was associated with cardiovascular comorbidity, corticosteroid uses and with the presence of complications. Sociodemographic characteristics of the patients were similar to the world population. The institution has a low prevalence of anastomotic dehiscence, global complications are comparable with international statistics.

## Introduction

Colorectal cancer (CRC) is among the most frequent neoplasms worldwide^1^. The incidence of colon, rectal and anus cancer in Colombia is of 12.2 per 100,000 men and 12.3 per 100,000 women, thus being the fourth and third most prevalent types of cancer in the Colombian population^2^.

Colon and rectal cancer are different entities, the two structures have different embryological origins, functions, irrigation and microbiota, and therefore these types of neoplasms have different etiologies and risk factors^3^. Rectal cancer comprises approximately 35% of all colorectal cancer cases in the European Union, its incidence is expected to increase in both sexes in the coming years^4^.

The management of rectal cancer as well as colon cancer will depend on its stage and the prognostic factors. Local excision (transanal endoscopic microsurgery or minimally invasive transanal surgery) can be used in early stage T1N0 tumors^4, 5^. Tumors in more advanced stages should be treated with radical resection that includes mesorectal excision due to the greater risk of recurrence and involvement of mesorectal nodules^4, 5^.

Currently, advanced rectal cancers receive multimodal treatments that are effective for the control of local and distant recurrence^4, 5^. In patients were lesions come in contact with sphincters, preservative techniques or abdominoperineal resections will be considered according to the case^5^.

The decision of the laparoscopic, open or transanal approach depends on the expertise of the surgeon, the stage and location of the cancer, as well as patient factors such as obesity or the presence of previous abdominal surgery^4, 6^. The main complications related to the procedure are anastomotic dehiscence and surgical site infection; followed by organized fistulas, contiguous organ injury, etc.^7, 8^.

Outcomes can be related to preoperative characteristics of the patient, including the extent of the disease, sex, age, among others^7^. It is essential to know the factors associated with the outcomes, complications, morbidity and mortality of patients undergoing rectal surgery in our local context. This study retrospectively analyzes patients undergoing rectal cancer surgery, describing the characteristics and outcomes in an important fourth-level institution in Colombia, during the period within 2013–2017.

## Methods

We designed a descriptive study of a retrospective cohort, by reviewing the electronic records of all patients between 2013 and 2017, of patients with a diagnosis of rectal cancer treated with laparoscopic anterior rectal resection with institutional follow-up over 18 years of age. This study was approved by the Ethics Committee of the Hospital Universitario Mayor Mederi (CIMED—Centro de investigaciones de Mederi) act number 11/2017, in view of the retrospective nature of the study and all the procedures being performed were part of the routine care and informed consent was waived by all the patients in their admission of all the patients to the hospital. For the statistical analysis, the SPSS V22 program was used (license of the Universidad del Rosario).

The sociodemographic variables were age and gender. The clinical variables analyzed in the study included anthropometric variables such as weight, height, and body mass index, ASA classification, and comorbidities. Regarding variables related to the tumor, the following were studied: the primary site, the histology, the number of lymph nodes resected, and the distance to the anal margin. Regarding surgical variables, we describe intraoperative bleeding, rates of conversion to open procedure, transfusion of blood products, resection margins, requirement of postoperative drain, endoanal tube, and length of hospital stay. Regarding follow-up, variables such as hospital stay, in-hospital death, and postoperative complications were explored. All methods were performed in accordance with the Declaration of Helsinki, and in follow of the guidelines of the Hospital Universitario Mayor Méderi, counting with the consent of the patients and of the ethics committee of the Hospital Universitario Mayor Méderi and the Rosario University, Bogotá, Colombia. All data generated or analyzed during this study are included in this published article as a supplementary file S1.

### Ethical approval

This study was approved by the Ethics Committee of the Hospital Universitario Mayor Mederi act number 11/2017, in view of the retrospective nature of the study and all the procedures being performed were part of the routine care and informed consent was waived by all the patients in their admission of all the patients to the hospital.

## Results

A sample of 133 patients, who underwent laparoscopic anterior rectal resection by the coloproctology service, was obtained in the aforementioned period, of these 60.9% were men and 39.1% were women; the percentage of patients under 45 years of age was 9.8%, between 46 and 64 years 39.8% and those over 65 years 50.4%, the median body mass index was 23.9 with a standard deviation of 4.5, data shown in Table 1.

**Table 1:** Characterization of patients diagnosed with rectal cancer who underwent surgery.

Variable	n (%)		
Age (years)	25–45	13 (9.8%)	
	46–64	53 (39.8%)	
	Over 65	67 (50.4%)	
Sex	Female: 52 (39.1%)		
	Male: 81 (60.9%)		
Height (cm)	Median: 163		
	SD: 9.2		
Weight (kg)	Median: 64		
	SD: 13.8		
BMI	Median: 23.9		
	SD: 4.5		
Location of the tumor	Upper rectum includes rectosigmoidal junction		41 (30.8%)
	Middle rectum		42 (31.6%)
	Lower rectum		48 (36.1%)
Distance from the tumor to the anal margin	Median		8.5
	SD:		5.9
Resection margins	R0		97 (75.8%)
	R1		10 (7.5%)
	R2		16 (12.5%)
Dissected nodes	Median		7
	SD:		7
	Min: 0		Max: 29
Intraoperative bleeding	Greater than 50 ml		5 (3.8%)
	Less than50ml		128 (96.2%)
Conversion to open procedure			8 (6%)
Transfusion of blood products in the previous 72 h up to 7 days after the intervention			13 (9.8%)
Drain			91 (68.4%)
Endoanal tube			14 (10.5%)

The most frequent tumor location was the lower rectum with 36.1%, the median distance from the tumor to the anal margin was 8.5 cm with a standard deviation of 5.9 cm, the median of positive nodes (pathological) obtained in surgical specimens was 7, with a standard deviation of 7, with a minimum value of 0 and a maximum value of 29 nodes. In 75.8% of the patients an R0 resection was obtained, the conversion rate to open procedure was 6%. Up to 10.5%^14^ of the patients used an endoanal tube (Table 2).

**Table 2:** In-hospital clinical outcomes related to surgical intervention.

Hospital stay (days)	Median 9.4	
	SD: 15	
Less than 7 days	91 (68.4%)	
Longer than 7 days	42 (31.6%)	
Neoadjuvant	84 (63.2%)	
Histopathology	Well differentiated	44 (34.6%)
	Moderately differentiated	74 (58.3%)
	Badly differentiated	9 (7.1%9
Complications	34 (25.6%)	
Fistula	1 (0.8%)	
Surgical wound infection	3 (2.3%)	
Respiratory	1 (1.5%)	
Intestinal obstruction	4 (3%)	
Ileus	16 (12%)	
Dehiscence	5 (3.8%)	
Stenosis	0	
Postoperative bleeding	1 (0.8%)	
Urethral/bladder injury	1 (0.8%)	
Evisceration/Hernia	1 (0.8%)	
Clavien Dindo	I	16 (12%)
	II	6 (4.5%)
	IIIa	1 (0.8%)
	IIIb	7 (5.3%)
	IVa	1 (0.8%)
	IVb	0
	V	4 (3%)
In-hospital mortality	4(3%)	

25% from all the patients showed some complications, the most frequent was ileus (12%), followed by anastomotic dehiscence documented in 5 cases (3.8%), independently defined from the fistula presentation, which we characterized as the documentation of an organized epithelialized tract, with the single case classified as an enterocutaneous fistula. The amount of severe complications (claven dindio IV and V) which lead to re-intervention and death were only 3.8%. In-hospital mortality was 3%; most of the patients had a short course in hospital stay (68%). About 75% of the patients had a R0 resection, in the follow-up, no more cases of mortality were documented.

## Discussion

Rectal cancer comprises approximately 35% of all colorectal cancer cases in the European Union, its incidence is expected to increase in both sexes in the coming years^4^. In developing countries or low-risk areas such as Asia, rectal cancer represents the highest number of all CRC (50% or more), unlike Europe and the United States where the incidence of rectal cancer among all CRC is generally less than 40%^3^.

Colon and rectal cancer are different entities, the two structures have different embryological origins, functions, irrigation and microbiota, therefore these types of neoplasms have different etiologies and risk factors^3^. It is estimated that 2 thirds of cases are associated with modifiable risk factors, such as high body mass index, the amount of intra-abdominal fat and type II diabetes, long-standing Crohn's disease affecting the rectum, excess consumption of red and processed meats, tobacco use and moderate or abundant alcohol consumption^4, 9, 10^. Protective factors such as the consumption of garlic, milk, calcium and abundant fiber diet^4, 10^. Within the population studied, it is evidenced that the age most affected by rectal cancer in the institution, corresponded to patients over 65 years of age, followed by the group of 46–64 years. This corresponds to the characterization of 2018 in high-cost accounts, identifying the highest number of cases in patients between 50 and 80 years of age with a 91% rate^5^. However, the current trend at the global level is that the incidence has begun to increase in patients under 50 years of age, apparently secondary to a change in lifestyle and an increase in screening^7, 8^.

Regarding sex, there was a higher incidence in men (60.9%) than in women (39.1%). Findings which are consistent with the statistics of America and Asia (85% in men and 79% in women). This is in contrast to the high-cost national data, where it is almost equivalent between the two sexes, with a slight superiority in the female group (44.71 vs 55.11%)^3^.

Among the variables of our study, being overweight was considered since it has been associated with a higher risk of presenting rectal cancer. In fact, in the study by Dong et al., It is emphasized that body mass index (BMI) may be a better predictor than obesity in general^11^.

Significant intraoperative bleeding (established as more than 50cc for the study) was only found in 3.8%, which is equivalent to only 5 patients. Life-threatening bleeding is one of the most unusual complications in colorectal surgery, with progressive decline over the years^12^. Conversion to an open procedure was found in 6% of the patients, which, compared to the study by Liu et al., was lower (8.4%). In our study, only 2 of the patients who required conversion to an open procedure had obesity with a BMI of 30 and 31 kg/m^2^, respectively. However, this value is consistent with international literature where the range of coverage can be from 0 to 25%^12^.

The distribution of rectal lesions in our study shows that up to 36.1% of the patients had a tumor classified as low, on the other hand, the median distance in centimeters from the tumor to the anal margin was 8.5 cm, high in relation to reports by other authors such as Fleshman who describe an average distance to the anal margin of 3.2 cm^13^, this data surely impacts the choice of other types of procedures such as abdominoperineal resections excluded in the methodology of our protocol.

The most frequent procedure in our department was a laparoscopic anterior resection of the rectum, routinely with a protective ileostomy, with a low percentage of conversion to open surgery generally associated with more advanced tumor stages, according to Pedziwiatr and cols, in a systematic review with 2018 patients with laparoscopic management and 1526 with an open approach, do not identify differences in positive circumferential margins (RR 1.16, 95% CI 0.89–1.50) or complete mesorectal excision (-0.01, 95% CI − 0.89 to 0.87), considering the two approaches equivalent in terms of oncological results^14–16^.

The dissection and procurement of nodes is very important, in our study the median of positive nodes obtained in the pathology sample was 7, with extreme data ranging from 0 nodes to 29, in relation to data obtained from the fund of high cost disease in Colombia in 2018, 75% of the pathology samples obtained had less than 12 nodes^2^, this suggests improvements in the treatment of the pathology sample and on the other hand, evaluation of the quality of the mesorectum obtained from the surgical specimen.

Approximately 70% of our patients had an average hospital stay of 7 days, data similar to that obtained by Fleshman et al., in a multicenter non-inferiority study between laparoscopic and open surgery which describes that the group of patients operated on with anterior rectal resection for laparoscopic had an average stay of 7.3 days^13^. The data is very similar to the vast majority of multicenter studies that describe good postoperative results with rectal surgery^13, 14^.

Maintaining a standardization of the surgical technique impacts predictable postoperative results. In our study, 25.6% of patients with global postoperative complications clearly show this trend, data which is similar to that obtained by Fleshman et al.^13^.

In a prospective study, Rickert obtains data from 276 consecutive patients operated on for rectal cancer and discriminates for laparoscopic surgery a cumulative risk percentage of postoperative ileus with and without reoperation of up to 25%, data well above that achieved in our institution, where the main postoperative complication was fixed at 12%^17^.

The percentage of anastomotic dehiscence is below the average reported in the international literature, our work obtained a 3.8% average, data concordant with work by Martin in 2013^12, 18^, which reports percentages ranging from 1.2 to 13% in anterior rectal resections by laparoscopy. The use of double staple suture in the creation of the anastomosis was an important factor associated with tumors located less than 10 cm and on the other hand a comparative study of no inferiority describes laparoscopic surgery as a protective factor for anastomotic dehiscence compared to open surgery (9.42% vs 13.47%)^18, 19^.

The comorbidity that marked an association with mortality in our study was cardiovascular comorbidity because of ith high prevalence, similar to that reported by Pellino G. et al., who documented this pathology in 52.8% of the patients in their study^19^.

## Conclusions

The sociodemographic characteristics of the patients operated on for rectal cancer at the institution are similar to the world population. The institution has a low rate of dehiscence and global complications compared to international statistics. Complete R0 dissection reduces patient morbidity and mortality in hospital. It is important to assess the causes of global mortality and through prospective studies explore the associated factors in greater depth to find opportunities for improvement. Recognizing the limitations of our study's methodology and sample size, we acknowledge the necessity of conducting more rigorous research with larger sample sizes to identify risk factors associated with the short-term outcomes described in our cohort. It is crucial to expand the scope of future studies to enhance our understanding of these factors and their implications for patient care.

### Supplementary Information

Supplementary Information.

## Data Availability

All data generated or analyzed during this study are included in this published article as a supplementary file S1.

## Abbreviations

CRC: Colorectal cancer
HUM: Hospital Universitario Mayor Mederi
BMI: Body mass index
Dx: Diagnosis
